# Treatment Outcomes in Undocumented Hispanic Immigrants with HIV Infection

**DOI:** 10.1371/journal.pone.0060022

**Published:** 2013-03-26

**Authors:** Kenneth K. Poon, Bich N. Dang, Jessica A. Davila, Christine Hartman, Thomas P. Giordano

**Affiliations:** 1 Department of Medicine, Baylor College of Medicine, Houston, Texas, United States of America; 2 VA Health Services Research and Development Center of Excellence, Michael E. DeBakey VA Medical Center, Houston, Texas, United States of America; Infectious Disease Service, United States of America

## Abstract

**Objective:**

Little is known about the treatment outcomes of undocumented Hispanic immigrants with HIV infection. We sought to compare the treatment outcomes of undocumented and documented patients 12-months after entering HIV care.

**Methods:**

We conducted a retrospective cohort study of antiretroviral-naive patients 18 years and older attending their first visit at Thomas Street Health Center in Houston, Texas, between 1/1/2003 and 6/30/2008. The study population of 1,620 HIV-infected adults included 186 undocumented Hispanic, 278 documented Hispanic, 986 Black, and 170 White patients. The main outcome measures were retention in care (quarter years with at least one completed HIV primary care provider visit) and HIV suppression (HIV RNA <400 copies/mL), both measured 12-months after entering HIV care.

**Results:**

Undocumented Hispanic patients had lower median initial CD_4_ cell count (132 cells/mm^3^) than documented Hispanic patients (166 cells/mm^3^; *P* = 0.186), Black patients (226 cells/mm^3^; *P*<0.001), and White patients (264 cells/mm^3^; *P* = 0.001). However, once in care, undocumented Hispanic patients did as well or better than their documented counterparts. One year after entering HIV care, undocumented Hispanics achieved similar rates of retention in care and HIV suppression as documented Hispanic and White patients. Of note, black patients were significantly less likely to have optimal retention in care (adjusted odds ratio [aOR] 0.65, CI = 0.45–0.94) or achieve HIV suppression (aOR 0.32, CI = 0.17–0.61) than undocumented Hispanics.

**Conclusions:**

Undocumented Hispanic persons with HIV infection enter care with more advanced disease than documented persons, suggesting testing and/or linkage to care efforts for this difficult-to-reach population need intensification. Once diagnosed, however, undocumented Hispanics have outcomes as good as or better than other racial/ethnic groups. Safety net providers for undocumented immigrants are vital for maintaining individual and public health.

## Introduction

HIV infection disproportionately affects Hispanics in the United States (US). While Hispanics represent about 16% of the total US population, they represent 20% of persons newly infected with HIV [1]–[4]. Furthermore, Hispanics are significantly more likely to enter HIV care with advanced disease [2], [5]–[8]. Entry into HIV care late in the disease process results in worse clinical outcomes, including lower rates of viral suppression and worse survival [9]–[12]. In a multi-site study of 1,357 treatment naïve patients, Hispanics had significantly shorter AIDS-free survival compared to non-Hispanic whites [13]. Differences in disease severity at initiation of antiretrovirals partly explained this observed variation. Hispanics represent a heterogeneous population, diverse in country of origin and residency status. While most of the 50.5 million Hispanics residing in the US are citizens and many have family roots in the US stretching back generations, an estimated 9.9 million lack legal documentation [14], [15]. To better understand the barriers to timely HIV care and uptake of antiretroviral therapy, studies need to account for this diversity.

Within the Hispanic population, immigrants, especially the undocumented, may be particularly vulnerable to barriers to regular HIV care. Of 75 foreign-born Hispanic patients attending a public health system in Atlanta, 69% were employed but only 17% had health insurance [16]. The majority had CD_4_ cell counts less than 200 cells/mm^3^ at the time of HIV diagnosis, and HIV testing most commonly occurred in the process of seeking treatment for an acute illness. Undocumented Hispanics may enter HIV care late because they have either experienced or perceived structural barriers to accessing health care. Findings from a recent qualitative study suggests that some undocumented Hispanic immigrants in Houston fear accessing publicly funded clinics due to deportation concerns or have difficulty navigating the health care system due to language barriers [17].

Although several studies report disparities in HIV care among different racial and ethnic groups, little is known about the effects of legal residency status on health outcomes. Following up on our qualitative research, we conducted a retrospective cohort study to compare the health outcomes of undocumented Hispanics and documented persons obtaining care in a large county HIV clinic in Texas [17].

## Methods

### Population

We conducted a retrospective cohort study of antiretroviral-naive patients attending their first visit at Thomas Street Health Center (TSHC) between 1/1/2003 and 6/30/2008. Thomas Street Health Center is a publicly funded, free-standing HIV clinic that provides care to residents of the Houston metropolitan area regardless of ability to pay. Proof of residency in the metropolitan area is required to receive care, but legal residency in the US is neither required nor queried. Patients presenting for care during this time frame were divided into four groups consisting of undocumented Hispanic, documented Hispanic, documented non-Hispanic Black (hereafter, Black), and documented non-Hispanic White (hereafter, White) persons. Self-reported race and ethnicity were abstracted from electronic medical and administrative databases.

Exclusion criteria included: 1) receipt of antiretroviral therapy before entry to TSHC; 2) Asian race; 3) non-Hispanic Black or White race with an invalid or no social security number (SSN); 4) no CD_4_ cell count or HIV viral load completed at intake to TSHC; 5) age less than 18 years; and 6) a baseline HIV viral load <400 copies/mL.

### Measures

#### Undocumented status

Data were obtained from electronic medical and administrative databases at TSHC. Patients with an invalid SSN or no SSN were classified as undocumented. Invalid SSNs are series that have never been assigned by the US Social Security Administration. For the SSN “XXX-YY-ZZZZ”, invalid series included: any combination containing XXX of 000 or 666, YY of 00, or ZZZZ of 0000. SSNs higher than 772-82-9999 were also invalid [18]. “No SSN” was defined as having a generically assigned 999-99-9999 series or no number in the TSHC administrative database.

#### Retention in HIV care

Retention in care during the first year was based on the number of 3-month quarters with at least one completed HIV primary care provider visit [19]. This measure has been shown to predict mortality in persons starting antiretroviral therapy [10]. Retention data were categorized as all 4 quarters with a visit (optimal retention), between 1 and 3 quarters with a visit, and no quarters with a visit.

#### HIV suppression

HIV suppression was defined as an HIV viral load of less than 400 copies/mL at one year ±90 days. The percentage of patients with HIV suppression at one year was measured only for those patients who had a baseline CD_4_ cell count <350 cells/mm^3^, the recommended threshold for initiating antiretroviral therapy during the time period of the study [20]. Since retention in care was a separate outcome, we did not use a “missing equals failure” approach in the viral suppression analysis, and instead only included persons with an HIV viral load in the medical record.

#### Other measures

Demographics data obtained include age, sex, HIV risk factors, marital status, income, absolute CD_4_ cell counts, HIV viral load, and appointment dates. HIV risk factors were categorized into the following exclusive groups: men who have sex with men (MSM), intravenous drug usage (IDU), IDU with MSM, heterosexual, and other. The IDU and IDU with MSM groups were combined due to the small numbers of patients reporting only IDU. Income data were gathered as categorical data relative to the federal poverty limit (FPL) for the patient’s household size. The CD_4_ cell count and HIV viral load at intake to TSHC and one year following the date of the intake visit (±90 days) were obtained.

### Statistical Analysis

Statistical analysis was performed using SAS 9.2 statistical software (SAS Institute, Cary, NC). Chi-squared tests were used to compare baseline characteristics. Baseline absolute CD_4_ cell count and log_10_ HIV viral load were compared with Kruskal-Wallis tests due to their nonparametric distribution. Univariate analyses compared outcomes for all four groups overall and for each group to the undocumented Hispanic group. Multivariate logistic regression was used to determine odds of optimal retention in care (visits in four of four quarters) and achieving HIV suppression at one year. Analyses were adjusted for age, sex, HIV risk factors, marital status, income relative to FPL, baseline absolute CD_4_ cell count <200 cells/mL, and baseline HIV viral load >10^5^ copies/mL. Additional multivariate analyses determined the odds of optimal retention in care and achieving HIV suppression among just the undocumented and documented Hispanic groups.

The study was approved by the Institutional Review Board of Baylor College of Medicine and Affiliated Hospitals. As the data were analyzed anonymously, the IRB granted a waiver of the requirement for individual consent.

## Results

**Figure 1:**
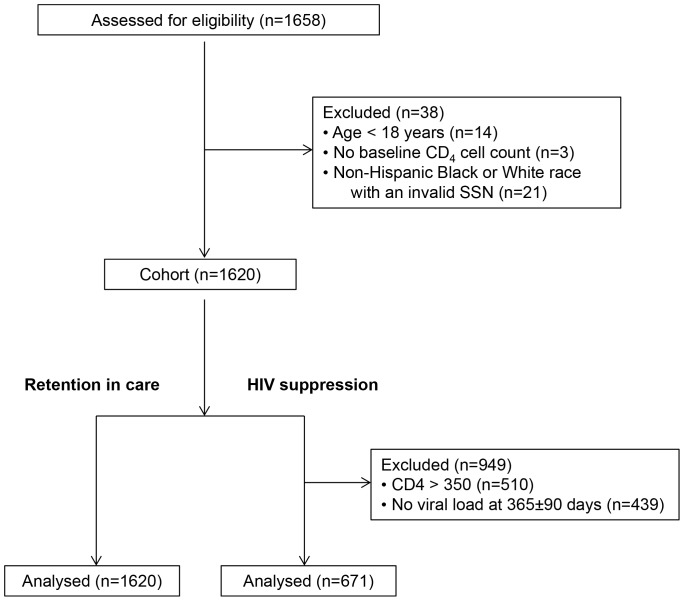
Flow Diagram.

A total of 1,658 new patients established care at TSHC between 01/01/2003 and 6/30/2008 (Figure 1). Patients excluded from analyses include 14 patients with age less than 18 years at intake, 3 patients with no baseline CD_4_ cell count results, and 18 Black and 3 White patients who had invalid SSNs. The final study population of 1,620 HIV-infected adults included 186 undocumented Hispanic, 278 documented Hispanic, 986 Black, and 170 White patients.

Demographic characteristics are shown in Table 1. There were significant differences in age, sex, marital status, income <100% FPL, and HIV risk factors between the groups. The undocumented Hispanic group was younger, had the lowest percentage of females, and had a higher percentage of married individuals than the Black and White groups. The undocumented group also had a higher percentage of monolingual Spanish speakers than the documented Hispanic group (62.4% versus 30.7%).

**Table 1:** Baseline characteristics of persons initiating care at Thomas Street Health Center in Houston, Texas.

Characteristics	Total	Undocumented Hispanic*	Documented Hispanic	Black	White	p**
	N = 1620	n = 186	n = 280	n = 984	n = 170	
Age, Median Years n(%)†, ‡, §						<0.0001
<30 years	399 (24.6)	73 (39.3)	65 (23.2)	232 (23.6)	29 (17.1)	
30–39 years	528 (32.5)	81 (43.6)	87 (31.1)	307 (31.2)	52 (30.6)	
40–49 years	473 (29.1)	25 (13.4)	74 (26.4)	312 (31.7)	62 (36.5)	
>50 years	221 (13.7)	7 (3.8)	54 (19.3)	133 (13.5)	27 (15.9)	
Female n(%)‡	508 (31.4)	40 (21.5)	62 (22.1)	360 (36.6)	46 (27.1)	<0.0001
Married n(%)‡, §,||	286 (19.5)	51 (27.7)	76 (29.8)	142 (16.1)	17 (11.3)	<0.0001
Monolingual-Spanish Speakers n(%)†, ‡, §	202 (12.4)	116 (62.4)	86 (30.7)	0 (0.0)	0 (0.0)	<0.0001
Income <100% Federal Poverty Level n(%)‡, #	1222 (76.9)	123 (69.5)	184 (66.0)	791 (81.6)	124 (75.2)	<0.0001
HIV Risk Factors n(%)†, ‡, §						<0.0001
MSM	439 (27.1)	58 (31.2)	101 (36.1)	210 (21.3)	70 (41.2)	
IDU and IDU+MSM	109 (6.7)	0 (0.0)	14 (5.0)	57 (5.8)	38 (22.4)	
Heterosexual	963 (59.4)	118 (63.4)	147 (52.5)	647 (65.8)	51 (30.0)	
Other	109 (6.7)	10 (5.4)	18 (6.4)	70 (7.1)	11 (6.5)	
Median CD4 cells/mm^3^ (IQR)‡, §	201 (48–413)	132 (37–308)	166 (47–383)	226 (49–439)	264 (84–504)	0.0001
Median Viral Load Log_10_ (IQR)†, ‡, §	5.08 (4.43–5.60)	5.40 (4.81–5.85)	5.14 (4.53–5.65)	5.00 (4.33–5.53)	5.05 (4.47–5.66)	<0.0001

* Undocumented defined as having an invalid social security number (SSN) or no SSN.

** = p value when comparing all groups in the analysis for each category.

† = p≤0.006 when comparing Undocumented Hispanic and Documented Hispanic groups.

‡ = p≤0.006 when comparing Undocumented Hispanic and Documented non-Hispanic Black groups.

§ = p≤0.006 when comparing Undocumented Hispanic and Documented non-Hispanic White groups.

|| = calculated for 1471; 152 patients did not have marriage information available in the database.

# = calculated for 1590; 30 patients did not have income information available in the database.

** = Undocumented individuals are those who do not have a valid social security number.

At baseline, the undocumented Hispanic group had the highest median log_10_ HIV viral load (5.40) and lowest median CD_4_ cell count (132 cells/mm^3^). Specifically, undocumented Hispanic patients had significantly lower CD_4_ cell counts than Black and White patients, and significantly higher log_10_ HIV viral load than documented Hispanic, Black, and White patients (Table 1).

Treatment outcomes according to racial ethnic groups are shown in Table 2. The undocumented and documented Hispanic groups had the most patients with optimal retention in care (37.6% and 38.6%). In pairwise comparisons (data not shown in Table 2), only the Black patients significantly differed in the distribution of quarterly visits from the undocumented Hispanic group, with 27.2% optimally retained in care (p = 0.01). The Black group also had a significantly lower percent achieving HIV suppression compared to the undocumented Hispanic group (58.8% versus 82.4%, p<0.0001). Treatment outcomes in the documented Hispanic and White groups did not differ significantly from the undocumented Hispanic group.

**Table 2:** Treatment outcomes one year after initiating care at Thomas Street Health Center in Houston, Texas.

Outcome	Total	Undocumented Hispanic*	Documented Hispanic	Black	White	p**
	N = 1620	n = 186	n = 280	n = 984	n = 170	
**Retention in care, n (%)** †						0.0008
Optimal, 4 quarters with visit	499 (30.8)	70 (37.6)	108 (38.6)	268 (27.2)	53 (31.2)	
1 to 3 quarters with visit	988 (61.0)	104 (55.9)	160 (57.1)	623 (63.3)	101 (59.4)	
No quarters with visit	133 (8.2)	12 (6.5)	12 (4.3)	93 (9.5)	16 (9.4)	
	N = 671	n = 85	n = 147	n = 387	n = 52	
**VL<400 c/mL, n(%)** †,‡	451 (67.2)	70 (82.4)	115 (78.2)	224 (58.8)	42 (80.8)	<0.0001

* Undocumented defined as having an invalid social security number (SSN) or no SSN;

** = p value comparing all groups.

† = p value was significant when comparing Undocumented Hispanic and Documented non-Hispanic Black groups.

‡ = calculated for patients who had absolute CD_4_ counts ≤350cells/mm^3^ at baseline and a recorded value at 365+/−90days.

§ = p values were not significant when comparing Undocumented Hispanic to Documented Hispanic or Documented non-Hispanic White groups.

The multivariate regression models of retention in care and HIV suppression at one year after initiating care at TSHC are shown in Table 3. For both outcomes, undocumented Hispanics did as well as documented Hispanic and White patients. Black participants were significantly less likely to have optimal retention in care (adjusted odds ratio [aOR] 0.65, CI = 0.45–0.94) or achieve HIV suppression (aOR 0.32, CI = 0.17–0.61). Patients with age >50 years, income >100% FPL, and baseline VL log_10_<5 were significantly more likely to have optimal retention in care (Table 3). Patients with age >50 years, married status, and income >100% FPL were also more likely to achieve HIV suppression. In separate regression models of both outcomes restricted to only Hispanic patients, adjusting for Spanish monolingualism did not change the lack of significant differences between the undocumented and documented groups (data not shown).

**Table 3:** Multivariable analysis of retention in care and HIV suppression one year after initiating care at Thomas Street Health Center in Houston, Texas.

Variables	Optimal retention in care* ^,^ †(odds ratio, 95% CI)	p	Achieved VL<400 copies/mL ‡(odds ratio, 95% CI)	p
Undocumented Hispanic**	Reference		Reference	
Documented Hispanic	0.93 (0.45–1.23)	0.73	0.69 (0.33–1.41)	0.30
Black	0.65 (0.45–0.94)	0.02	0.32 (0.17–0.61)	0.0004
White	0.74 (0.45–1.23)	0.25	0.95 (0.35–2.59)	0.91
Age <30 years	Reference		Reference	
Age 30–39 years	0.85 (0.63–1.16)	0.30	1.48 (0.91–2.38)	0.11
Age 40–49 years	1.14 (0.83–1.57)	0.41	1.58 (0.95–2.65)	0.08
Age >50 years	1.38 (1.38–3.16)	0.0005	2.49 (1.21–5.13)	0.01
Female	Reference		Reference	
Male	1.17 (0.88–1.56)	0.29	1.15 (0.73–1.81)	0.54
Single	Reference		Reference	
Married §	0.91 (0.67–1.25)	0.58	1.84 (1.09–3.08)	0.02
Income <100% FPL	Reference		Reference	
Income >100% FPL ||	2.08 (1.59–2.71)	<0.0001	1.98 (1.25–3.13)	0.004
Heterosexual	Reference		Reference	
MSM	1.04 (0.76–1.41)	0.81	1.14 (0.70–1.87)	0.60
IDU+Other	1.19 (0.83–1.70)	0.35	0.91 (0.52–1.61)	0.75
Baseline Absolute CD_4_>200	Reference		Reference	
Baseline Absolute CD_4_<200	1.21 (0.93–1.57)	0.17	1.57 (1.01–2.43)	0.05
Baseline Viral Load Log_10_>5	Reference		Reference	
Baseline Viral Load Log_10_<5	1.41 (1.09–1.84)	0.01	1.13 (0.74–1.70)	0.58

* Optimal Retention in care is defined as having fulfilled an appointment with an HIV care physician in all 4 quarters.

** Undocumented defined as having an invalid social security number (SSN) or no SSN.

† = Total N = 1620.

‡ = calculated for 671.

§ = calculated for 1471; 152 patients did not have marital status information available in the database.

|| = calculated for 1590; 30 patients did not have income information available in the database.

## Discussion

Undocumented residency status is distinct from immigration status, race, and ethnicity, for many Hispanics are multi-generational US citizens, while others are legal residents and immigrants. Previous studies have not made the distinction between documented and undocumented and group all Hispanic patients together, potentially obscuring differences associated with legal status [5], [6], [8], [13], [16], [21]–[24]. In this retrospective cohort study of 1,620 antiretroviral-naive patients who established HIV care at TSHC from 2003 to 2008, undocumented Hispanic patients presented with the most advanced HIV disease. However, once in care, undocumented Hispanic patients did as well or better than their documented counterparts. One year after entering HIV care, undocumented Hispanics achieved similar rates of retention in care and HIV suppression as documented Hispanic and White patients. Additionally, they had more favorable retention and treatment outcomes than Black patients.

Undocumented persons often endure formidable journeys to cross the US border and oftentimes work in physically demanding jobs once in the US [25]. Their sense of resilience and self-efficacy most likely differ from their native-born counterparts in the countries from which and to which they migrated. Studies have consistently shown more favorable health outcomes in foreign-born Hispanic patients and use terms such as the “healthy migrant effect” and “Hispanic paradox” in explaining this phenomenon [26]. Possible explanations include selective migration, less adverse health behaviors, and greater social support. Indeed, the undocumented Hispanic patients in our study were younger than the other racial/ethnic groups, and Hispanics were more often married. In some cases, marital status may serve as a proxy for positive social support [27]. Undocumented Hispanic patients were also significantly less likely to have an income <100% FPL than documented Black and White patients. These data are consistent with studies showing that most undocumented immigrants enter the US primarily to find employment opportunities and work [28], [29].

Undocumented Hispanic patients entered care later and with more advanced disease than other groups. Our findings are consistent with previous studies showing minorities, including Hispanic patients, entering care later than White patients [5], [8], [9], [16], [22], [30]–[33]. Possible explanations for delays in care include lack of health insurance, lack of a regular source of care, different cultural and societal perceptions of health, competing subsistence needs, and fear of legal ramifications regarding citizenship if they present for medical care, especially at public healthcare facilities [16], [29], [30], [33]–[42].

Although undocumented Hispanic patients accessed HIV care later compared to Black and White patients, their retention in care and rate of HIV suppression were similar or better at one year. Our data suggests that after entering care, undocumented Hispanic patients were more likely to be retained in care. This differs from some studies showing Hispanic patients having low continuity of care [21]; however, it is consistent with other studies showing Hispanic individuals having similar or better retention in care than White or Black individuals [6], [24]. Of note, neither our study nor the studies cited above distinguished country of origin, which has been associated with HIV/AIDS outcomes [23].

Despite reported differences in healthcare access and utilization between undocumented and documented Hispanic immigrants [43], our study found no significant difference in treatment outcomes between the two groups. The barriers associated with undocumentedness may have been offset by characteristics specific to our patient population, clinic, and/or surrounding community. In-depth qualitative and prospective quantitative studies utilizing objective markers of legal residency status are needed to examine potential differences between the undocumented and documented populations.

Our study results indicate that publicly funded health care systems, like TSHC, serve as effective safety net providers in providing HIV care for undocumented Hispanic immigrants with HIV infection. Though the undocumented may have difficulty accessing some federal and state insurance programs such as Medicare and Medicaid, other programs such as locally funded programs, Ryan White grant funded programs, state AIDS Drug Assistance Programs (ADAP), and Section 330 funded Federally Qualified Health Centers are also available regardless of legal residency status [44]. TSHC is a county-funded clinic that receives Ryan White funds, and its patients benefit tremendously from the Texas ADAP program. Our findings suggest that these programs serving persons with HIV improve individual health and may be protecting the public’s health by reducing HIV viral load in an important, difficult-to-reach population.

While our study focuses on treatment outcomes in undocumented Hispanics, it is important to note that documented Black patients had significantly worse HIV outcomes than all other racial ethnic groups. The reasons for this disparity are not immediately apparent. Although Black patients were significantly less likely to be married or have an income >100% FPL, our analyses adjusted for these differences. However, our measure of income may have been inadequate and it is possible that undocumented Hispanics may have underreported their income due to fear of legal ramifications. Moreover, this study took place at a public clinic. With documented Black patients, a selection bias may exist, as those who get care at this clinic may differ significantly from Black patients who get care at non-public HIV clinics. They may represent those who have the most barriers to medical care. In contrast, undocumented Hispanics have fewer clinic options and selection bias, if any, would play less of a role. Differences in HIV outcomes could also be due to unmeasured confounders, such as psychosocial, structural, and environmental factors. Possible factors include depression, substance use, less social support, greater unmet need (e.g. housing and transportation), and residence in areas with high rates of crime and incarceration) [45], [46]. These factors, especially when juxtaposed against the “healthy migrant effect,” may play a role in influencing pathways through which racial disparities in HIV outcomes occur. Data have shown that even in a randomized controlled trial, where patients presumably have good access to care and clinical support, Black patients had worse HIV outcomes [13]. Thirty years into the epidemic, core questions remain regarding the best approaches to developing interventions that respond to the specific needs of minorities groups disproportionately affected by HIV/AIDS.

A methodological strength of this study includes the use of an innovative objective marker of legal residency status to determine undocumentedness. Among studies examining the effects of citizenship and legal residency on access to insurance, health care, and health status, none used objective markers for legal residency [33], [34], [40]–[42]. Self-report of residency status may be inaccurate due to fears of legal consequences. The use of SSNs to categorize documentedness creates an objective marker that may decrease misclassification bias due to distrust resulting from direct inquiry of residency status.

This study has certain limitations. The retrospective nature of the study limits the ability to explore socio-psycho-behavioral factors that could explain the differences in treatment outcomes. Additionally, the use of SSNs to determine legal residency status may result in conservative estimates of the true undocumented patient population. Patients lacking legal residency status may use a valid SSN issued under a different person’s name or a SSN issued under his or her name but associated with documents that have since expired (i.e., an immigrant with an expired visa has a valid SSN issued under his or her name). The study is a single-site study from Texas, and results may not generalize to the US population. Finally, neither documented nor undocumented Hispanics are homogenous populations. Nor, for that matter, are Black and White populations. We were unable to assess level of acculturation in any of the groups studied.

### Conclusions

Undocumented Hispanic patients are more likely to enter HIV care later and with concurrent AIDS than other populations. Once in care though, they have treatment outcomes as good as or better than their documented counterparts. These findings indicate that publicly funded health care systems, like TSHC, that receive federal, state and local funds to care for patients regardless of legal residency status or ability to pay do improve the individual and public health. However, the high number of undocumented Hispanic patients entering care with advanced HIV disease calls for intensified efforts to promote early HIV testing and linkage in care for this difficult-to-reach population.

## References

[1] Centers for Disease Control and Prevention (2012) HIV surveillance report, 2010; vol. 22. http://www.cdc.gov/hiv/topics/surveillance/resources/reports. Accessed February 11, 2013.

[2] Centers for Disease Control and Prevention (2010) Estimated lifetime risk for diagnosis of HIV infection among Hispanics/Latinos –37 states and Puerto Rico, 2007. MMWR Morb Mortal Wkly Rep 59(40): 1297–301. 20948507

[3] Prejean J, Song R, Hernandez A, Ziebell R, Green T, et al (2011) Estimated HIV incidence in the United States, 2006–2009. PLoS One 6(8): e17502. 21826193 10.1371/journal.pone.0017502 PMC3149556

[4] U.S. Census Bureau. State and county QuickFacts. Available at: http://quickfacts.census.gov/qfd/states/00000.html. Accessed December 3, 2012.

[5] Swindells S, Cobos DG, Lee N, Lien EA, Fitzgerald AP, et al (2002) Racial/ethnic differences in CD4 T cell count and viral load at presentation for medical care and in follow-up after HIV-1 infection. AIDS 16(13): 1832–4. 12218399 10.1097/00002030-200209060-00020

[6] Torian LV, Wiewel EW, Liu KL, Sackoff JE, Frieden TR (2008) Risk factors for delayed initiation of medical care after diagnosis of human immunodeficiency virus. Arch Intern Med 168(11): 1181–7. 18541826 10.1001/archinte.168.11.1181

[7] Centers for Disease Control and Prevention (2009) Late HIV testing - 34 states, 1996–2005. MMWR Morb Mortal Wkly Rep 58(24): 661–5. 19553901

[8] Dennis AM, Napravnik S, Sena AC, Eron JJ (2011) Late entry to HIV care among Latinos compared with non-Latinos in a southeastern US cohort. Clin Infect Dis 53(5): 480–7. 21844031 10.1093/cid/cir434 PMC3156142

[9] Lucas GM, Chaisson RE, Moore RD (1999) Highly active antiretroviral therapy in a large urban clinic: Risk factors for virologic failure and adverse drug reactions. Ann Intern Med 131(2): 81–7. 10419445 10.7326/0003-4819-131-2-199907200-00002

[10] Giordano TP, Gifford AL, White AC Jr, Suarez-Almazor ME, Rabeneck L, et al (2007) Retention in care: A challenge to survival with HIV infection. Clin Infect Dis 44(11): 1493–9. 17479948 10.1086/516778

[11] Mugavero MJ, Lin HY, Willig JH, Westfall AO, Ulett KB, et al (2009) Missed visits and mortality among patients establishing initial outpatient HIV treatment. Clin Infect Dis 48(2): 248–56. 19072715 10.1086/595705 PMC2737584

[12] Mugavero MJ, Amico KR, Westfall AO, Crane HM, Zinski A, et al (2012) Early retention in HIV care and viral load suppression: Implications for a test and treat approach to HIV prevention. J Acquir Immune Defic Syndr 59(1): 86–93. 21937921 10.1097/QAI.0b013e318236f7d2 PMC3237801

[13] Giordano TP, Bartsch G, Zhang Y, Tedaldi E, Absalon J, et al (2010) Disparities in outcomes for African American and Latino subjects in the flexible initial retrovirus suppressive therapies (FIRST) trial. AIDS Patient Care STDS 24(5): 287–95. 20438378 10.1089/apc.2009.0332 PMC2933555

[14] Passel JS, Cohn D (2010) U.S. unauthorized immigration flows are down sharply since mid-decade. Washington D.C.: Pew Hispanic Center.

[15] Ennis SR, Ríos-Vargas M, Albert NG (2011) The Hispanic Population: 2010. 2010 Census Briefs; C2010BR-04 (Washington, DC: U.S. Census Bureau).

[16] Kelley CF, Hernandez-Ramos I, Franco-Paredes C, del Rio C (2007) Clinical, epidemiologic characteristics of foreign-born Latinos with HIV/AIDS at an urban HIV clinic. AIDS Read 17(2): 73–4, 78–80, 85–8. 17323506

[17] Dang BN, Giordano TP, Kim JH (2012) Sociocultural and structural barriers to care among undocumented Latino immigrants with HIV infection. J Immigr Minor Health 14(1): 124–31. 22012476 10.1007/s10903-011-9542-x

[18] Social security number verification service: High group list and other ways to determine if an SSN is valid. Available: http://www.ssa.gov/employer/ssnvhighgroup.htm. Accessed February 11, 2013.

[19] Mugavero MJ, Davila JA, Nevin CR, Giordano TP (2010) From access to engagement: Measuring retention in outpatient HIV clinical care. AIDS Patient Care STDS 24(10): 607–13. 20858055 10.1089/apc.2010.0086 PMC2965698

[20] Panel on Antiretroviral Guidelines for Adults and Adolescents. Guidelines for the use of antiretroviral agents in HIV-1-infected adults and adolescents. Department of Health and Human Services. November 3, 2008; 1–139. Available: http://aidsinfo.nih.gov/guidelines/archive/adult-and-adolescent-guidelines. Accessed February 11, 2013.

[21] Shapiro MF, Morton SC, McCaffrey DF, Senterfitt JW, Fleishman JA, et al (1999) Variations in the care of HIV-infected adults in the United States: Results from the HIV cost and services utilization study. JAMA 281(24): 2305–15. 10386555 10.1001/jama.281.24.2305

[22] Turner BJ, Cunningham WE, Duan N, Andersen RM, Shapiro MF, et al (2000) Delayed medical care after diagnosis in a US national probability sample of persons infected with human immunodeficiency virus. Arch Intern Med 160(17): 2614–22. 10999975 10.1001/archinte.160.17.2614

[23] Espinoza L, Hall HI, Hu X (2012) Diagnoses of HIV infection among Hispanics/Latinos in 40 states and Puerto Rico, 2006–2009. J Acquir Immune Defic Syndr 60(2): 205–13. 22334071 10.1097/QAI.0b013e31824d9a29

[24] Yehia BR, Fleishman JA, Metlay JP, Korthuis PT, Agwu AL, et al (2012) Comparing different measures of retention in outpatient HIV care. AIDS 26(9): 1131–9. 22382143 10.1097/QAD.0b013e3283528afa PMC3355231

[25] DeLuca LA, McEwen MM, Keim SM (2010) United States-Mexico border crossing: Experiences and risk perceptions of undocumented male immigrants. J Immigr Minor Health 12(1): 113–23. 18850270 10.1007/s10903-008-9197-4

[26] Franzini L, Ribble JC, Keddie AM (2001) Understanding the Hispanic paradox. Ethn Dis 11(3): 496–518. 11572416

[27] Sherbourne CD, Hays RD (1990) Marital status, social support, and health transitions in chronic disease patients. J Health Soc Behav 31(4): 328–43. 2135935

[28] Passel JS, Cohn D (2009) A portrait of unauthorized immigrants in the United States. Washington D.C.: Pew Hispanic Center.

[29] Berk ML, Schur CL, Chavez LR, Frankel M (2000) Health care use among undocumented Latino immigrants. Health Aff (Millwood) 19(4): 51–64. 10916960 10.1377/hlthaff.19.4.51

[30] Kang E, Rapkin BD, Springer C, Kim JH (2003) The “demon plague” and access to care among Asian undocumented immigrants living with HIV disease in New York City. J Immigr Health 5(2): 49–58. 14512758 10.1023/a:1022999507903

[31] Saracino A, El-Hamad I, Prato R, Cibelli DC, Tartaglia A, et al (2005) Access to HAART in HIV-infected immigrants: A retrospective multicenter Italian study. AIDS Patient Care STDS 19(9): 599–606. 16164386 10.1089/apc.2005.19.599

[32] Thrasher AD, Earp JA, Golin CE, Zimmer CR (2008) Discrimination, distrust, and racial/ethnic disparities in antiretroviral therapy adherence among a national sample of HIV-infected patients. J Acquir Immune Defic Syndr 49(1): 84–93. 18667919 10.1097/QAI.0b013e3181845589

[33] Pivnick A, Jacobson A, Blank AE, Villegas M (2010) Accessing primary care: HIV+ Caribbean immigrants in the Bronx. J Immigr Minor Health 12(4): 496–505. 19784773 10.1007/s10903-009-9293-0

[34] Hubbell FA, Waitzkin H, Mishra SI, Dombrink J, Chavez LR (1991) Access to medical care for documented and undocumented Latinos in a southern California county. West J Med 154(4): 414–7. 1877182 PMC1002788

[35] Cunningham WE, Andersen RM, Katz MH, Stein MD, Turner BJ, et al (1999) The impact of competing subsistence needs and barriers on access to medical care for persons with human immunodeficiency virus receiving care in the United States. Med Care 37(12): 1270–81. 10599608 10.1097/00005650-199912000-00010

[36] Miller JE, Guarnaccia PJ, Fasina A (2002) AIDS knowledge among Latinos: The roles of language, culture, and socioeconomic status. J Immigr Health 4(2): 63–72. 16228761 10.1023/A:1014542324401

[37] Kullgren JT (2003) Restrictions on undocumented immigrants’ access to health services: The public health implications of welfare reform. Am J Public Health 93(10): 1630–3. 14534212 10.2105/ajph.93.10.1630 PMC1448024

[38] Shedlin MG, Decena CU, Oliver-Velez D (2005) Initial acculturation and HIV risk among new Hispanic immigrants. J Natl Med Assoc 97(7 Suppl): 32S–7S. PMC2640649 16080455

[39] Loue S (2006) Preventing HIV, eliminating disparities among Hispanics in the United States. J Immigr Minor Health 8(4): 313–8. 16944327 10.1007/s10903-006-9001-2

[40] Ortega AN, Fang H, Perez VH, Rizzo JA, Carter-Pokras O, et al (2007) Health care access, use of services, and experiences among undocumented Mexicans and other Latinos. Arch Intern Med 167(21): 2354–60. 18039995 10.1001/archinte.167.21.2354

[41] Nandi A, Galea S, Lopez G, Nandi V, Strongarone S, et al (2008) Access to and use of health services among undocumented Mexican immigrants in a US urban area. Am J Public Health 98(11): 2011–20. 18172155 10.2105/AJPH.2006.096222 PMC2636432

[42] Rodriguez MA, Bustamante AV, Ang A (2009) Perceived quality of care, receipt of preventive care, and usual source of health care among undocumented and other Latinos. J Gen Intern Med 24 Suppl 3508–13. 19841999 10.1007/s11606-009-1098-2 PMC2764043

[43] Vargas Bustamante A, Fang H, Garza J, Carter-Pokras O, Wallace SP, et al (2012) Variations in healthcare access and utilization among Mexican immigrants: The role of documentation status. J Immigr Minor Health 14(1): 146–55. 20972853 10.1007/s10903-010-9406-9 PMC3256312

[44] US Department of Health and Human Services, Health Resources and Services Administration. The health center program: Section 330 of the public health service act (42 USCS § 254b): Authorizing legislation for the health center program. Available: http://bphc.hrsa.gov/policiesregulations/legislation/index.html. Accessed February 11, 2013.

[45] Smith KY (2009) Paying the price for late starts and early stops: Racial and sex disparities in HIV-related mortality. Clin Infect Dis 49(10): 1579–81. 19845470 10.1086/644773

[46] Stone VE (2012) HIV/AIDS in women and racial/ethnic minorities in the U.S. Curr Infect Dis Rep. 14(1): 53–60. 10.1007/s11908-011-0226-4 22139589

